# Repetitive Scuba Diving as a Novel Trigger for Diabetes Insipidus: A Case Report

**DOI:** 10.1016/j.ekir.2026.106536

**Published:** 2026-04-09

**Authors:** Billy Zeng, Sijie Zheng

**Affiliations:** 1 Division of Nephrology, University of California, San Francisco, USA; 2 Department of Nephrology, Kaiser Permanente East Bay, Oakland, California, USA

**To The Editor:**

Central diabetes insipidus (CDI) results from a lack of antidiuretic hormone, leading to excessive thirst and polyuria.^1^ Known etiologies include trauma, tumors, autoimmunity, and hypoxia.^2^ Scuba diving involves exposure to high pressure and hypoxic conditions, and is known to cause neurological complications like myelopathy and stroke.^3^ However, scuba diving has never been reported as a trigger for CDI. We present a novel case of a diver developing CDI, highlighting potential mechanisms of diving-induced pituitary damage.

A female in her 50s presented with a month of acute-onset polyuria and polydipsia. She drinks 3 liters of water daily with hourly urination, resulting in severe nocturia. Her symptoms began after an annual scuba diving vacation. She dived daily to depths of 100 feet for 2 weeks, an annual routine for 20 years. Her physical examination was unremarkable, medications did not include any drugs associated with CDI, and she had no lithium exposure.

A 24-hour urine collection exceeded 2.8 liters. Baseline laboratory tests showed a serum sodium of 145 mEq/l and low urine osmolality of 188 mOsm/kg (Supplementary Tables S1 and S2). A brain magnetic resonance imaging revealed an absent neurohypophysis on T1-sequence confirming structural pituitary dysfunction (Supplementary Figure S1). A previous head magnetic resonance imaging 20 years ago for head trauma was normal. She was empirically started on 0.1 mg of desmopressin (DDAVP, Ferring Pharmaceuticals, Inc., Parsippany, New Jersey) twice daily. After a single dose, her nocturia improved. Maintenance DDAVP stabilized her urine osmolality at 515 mOsm/kg (Supplementary Tables S1 and S2).Water deprivation test was done consistent with partial CDI (Figure 1).

**Figure 1:**
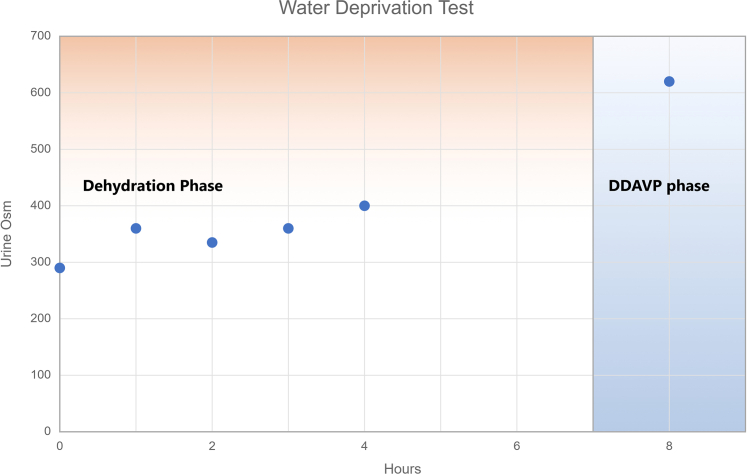
Water deprivation test.

This report details a novel association between repetitive scuba diving and CDI. The precise mechanism remains speculative; however, symptom onset after 20 years of identical diving suggests a threshold phenomenon, where antidiuretic hormone reserve was maintained until residual functional cell mass fell below a critical level. Although remote trauma or microvascular disease cannot be entirely discounted, her intensive diving is the most proximal trigger. Although our diagnosis relied on empirical desmopressin, emerging and highly accurate assays such as the copeptin assay may improve the future detection of such elusive CDI cases. (Supplementary Table S3)^4^^,^^5^

## Disclosure

SZ served on the scientific advisory board of Calliditas. BZ declares no competing interests.

## Patient Consent

Consent was obtained from the patient and filed under her medical records. Institutional approval was granted under RDO KPNC 25 – 396.

## Footnotes

Supplementary file (PDF). **Figure S1.** MRI showing an absence of neurohypophysis on T1 sequence as indicated by absence of bright spot in the pituitary gland. **Table S1.** Electrolytes at baseline, presentation, water deprivation, and maintenance desmopressin. **Table S2.** Urine osmolality at presentation, water deprivation test, and maintenance desmopressin. **Table S3.** Comparison of water deprivation test versus copeptin-based test.
