# Supplementary material for: Repetitive Scuba Diving as a Novel Trigger for Diabetes Insipidus: A Case Report
Source: Kidney Int Rep. 2026 Apr 9;11(6):106536. doi: 10.1016/j.ekir.2026.106536 (PMC13185904; doi:10.1016/j.ekir.2026.106536)
Supplement: Supplementary Material [file mmc1.pdf]

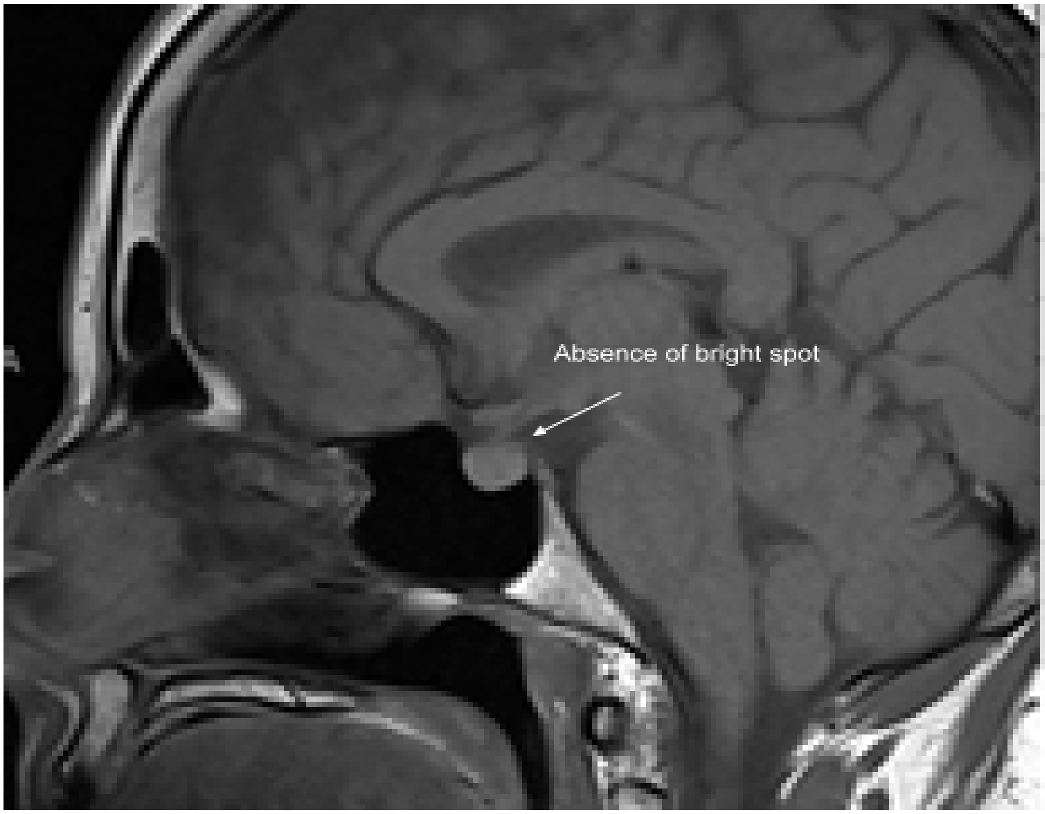

Supplemental Figure 1. MRI showing an absence of neurohypophysis on T1 sequence as indicated by absence of bright spot in the pituitary gland.

Supplemental Table 1: Electrolytes at Baseline, Presentation, Water Deprivation and Maintenance DDAVP

	1 year prior	Presentation		Water Deprivation Onset	Maintenance DDAVP			
Na	139	145	143	146	136	140	136	140
K	3.9	4.6	5.1		4.2	4.1	4.3	
Cl	103	106	103		99	103	100	
CO2	25	29	27		25	28	27	
Anion Gap	11						9	
BUN	18	8	16		16	16	14	
Glucose	109	103	92		93	96	93	
Creatinine	0.71	0.86	0.89		0.81	0.77	0.76	
eGFR		>60	>60		>60	>60	>60	

Supplemental Table 2: Urine Osmolality at Presentation, Water Deprivation Test, Maintenance DDAVP

	Presentation	Empiric DDAVP	Water Deprivation	Maintenance DDAVP		
OSMO UR	188	363	623<br>547<br>403<br>362<br>336<br>360<br>289	522	476	515

Supplemental Table 3. Comparison of Water Deprivation Test vs. Copeptin-Based Test

**Feature**	**Water Deprivation Test**	**Copeptin-Based Test**
**Principle**	Measures response to dehydration and desmopressin to assess vasopressin activity	Measures plasma copeptin (a stable surrogate for vasopressin) after hypertonic saline infusion
**Procedure Duration**	6–8 hours or longer	2–3 hours
**Patient Tolerance**	Poor; causes discomfort due to prolonged dehydration	Better tolerated; avoids prolonged fluid restriction
**Diagnostic Accuracy**	~76.6% overall accuracy	~96.5% overall accuracy with copeptin >4.9 pmol/L
**Risk of Complications**	Risk of desmopressin-induced hyponatremia, dehydration	Minimal; hypertonic saline infusion monitored closely
**Biomarker Used**	Indirect assessment of vasopressin via urine osmolality	Direct measurement of copeptin (C-terminal part of vasopressin precursor)

Feature	Water Deprivation Test	Copeptin-Based Test
**Clinical Utility**	Traditional gold standard but technically challenging	Emerging preferred method; more reproducible and patient-friendly
**Cost & Accessibility**	Low lab cost: high labor cost, requires nursing	Requires specialized lab for copeptin assay; higher lab cost
**Referenced Studies**	[4]	[5]
